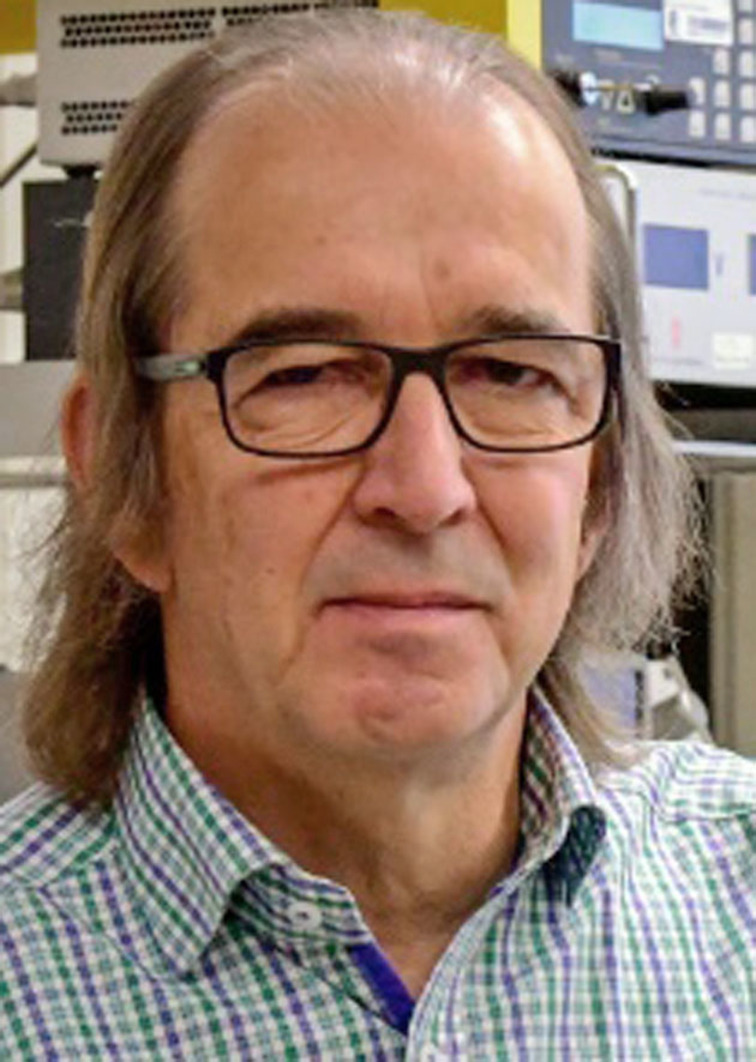# Improving Reproducibility in Hydrogen Storage Material Research

**DOI:** 10.1002/cphc.202100508

**Published:** 2021-08-31

**Authors:** Darren P. Broom, Michael Hirscher

**Affiliations:** ^1^ Max Planck Institute for Intelligent Systems Heisenbergstrasse 3 70569 Stuttgart Germany; ^2^ Hiden Isochema Ltd 422 Europa Boulevard Warrington WA5 7TS UK

**Keywords:** hydrogen, reproducibility, adsorption, metal hydrides, materials characterisation

## Abstract

Research into new reversible hydrogen storage materials has the potential to help accelerate the transition to a hydrogen economy. The discovery of an efficient and cost‐effective method of safely storing hydrogen would revolutionise its use as a sustainable energy carrier. Accurately measuring storage capacities – particularly of novel nanomaterials – has however proved challenging, and progress is being hindered by ongoing problems with reproducibility. Various metal and complex hydrides are being investigated, together with nanoporous adsorbents such as carbons, metal‐organic frameworks and microporous organic polymers. The hydrogen storage properties of these materials are commonly determined using either the manometric (or Sieverts) technique or gravimetric methods, but both approaches are prone to significant error, if not performed with great care. Although commercial manometric and gravimetric instruments are widely available, they must be operated with an awareness of the limits of their applicability and the error sources inherent to the measurement techniques. This article therefore describes the measurement of hydrogen sorption and covers the required experimental procedures, aspects of troubleshooting and recommended reporting guidelines, with a view of helping improve reproducibility in experimental hydrogen storage material research.

## Graphical abstract

**Accurately characterising the hydrogen sorption properties** of materials is critical to improving reproducibility in hydrogen storage material research. This review covers the main techniques used for this purpose, including important aspects of troubleshooting during the measurement process. It also proposes reporting guidelines for hydrogen sorption data, with the aim of improving reproducibility in the field of hydrogen storage material research.

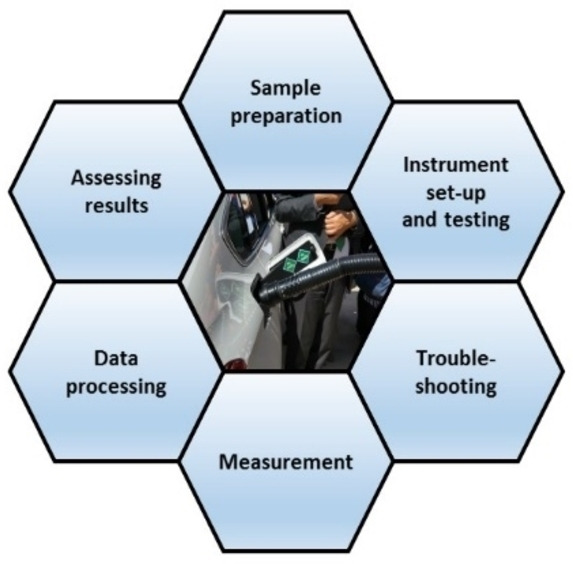

## 1 Introduction

Green hydrogen, produced from renewable energy, is set to play a pivotal role in the future transition to a low carbon energy system.^[1]^ Several countries now have national hydrogen strategies and, in 2017, the Hydrogen Council, a global consortium of companies – currently consisting of 109 members – was formed to help accelerate the worldwide development of hydrogen energy technology.^[2]^ In the envisioned scenario, hydrogen will act as both the principal energy carrier and a long term storage medium, for which it has significant advantages over competing energy storage technologies. For mobile applications, it is particularly effective as a sustainable energy carrier, because it contains a large amount of chemical energy per unit mass (142 MJ kg^−1^) and, when combined with O_2_ in a fuel cell, produces solely water. However, it is difficult to store safely in a compact and affordable form.[^3^, ^4^] One of the most promising solutions to this problem is the use of solid state hydrogen storage.[^5^, ^6^]

The earliest report of hydrogen absorption by a solid was the work on PdH_*x*_ by Thomas Graham in 1866.^[7]^ Palladium, however, is expensive and has a low gravimetric storage density of ∼0.6 wt.%. Other, cheaper, elemental metals have higher capacities, such as Ti (4 wt.%) and Mg (7.6 wt.%), but they have relatively high absorption and desorption temperatures, which is a major drawback, particularly for mobile storage applications. MgH_2_, for example, requires temperatures exceeding 570 K in order to reversibly store hydrogen. Many intermetallics, formed from a hydride‐forming element, A, and a non‐hydride‐forming element, B, are now known, some of which can operate at near ambient temperatures and pressures.[^8^, ^9^] The first to be reported, in 1958,^[10]^ was the AB compound ZrNi, which forms ZrNiH_3_, but a number of other families, including AB_5_ (e. g. LaNi_5_) and AB_2_ Laves phase compounds, have since been discovered.[^11^, ^12^] These materials have impressive volumetric storage capacities, which can exceed that of liquid H_2_,^[12]^ but their gravimetric capacities are generally below ∼2 wt.%.

In the 1990s, the automotive industry began in earnest to develop hydrogen fuel cell technology. This led to a marked increase in the investigation of new storage materials, including various complex hydrides. In 1997, Bogdanović and Schwickardi showed that Ti‐doping can lower the dehydriding temperature of sodium alanate (NaAlH_4_) to 420 K, and provide a reversible storage capacity of 5.6 wt.%.^[13]^ This was one of the major breakthroughs made during this period, together with later work by Chen and co‐workers on the reversible storage of hydrogen using the Li−N−H system, which has a potential gravimetric capacity of over 10 wt.%.^[14]^ Since then, the field of complex hydrides has grown considerably, with many new compounds reported.[^15^, ^16^, ^17^, ^18^]

Nanoporous adsorbents, meanwhile – including different types of carbon, and various zeolites, metal‐organic frameworks (MOFs), and microporous organic polymers – have also attracted widespread interest. These are high surface area materials with narrow pores that can physisorb large quantities of molecular hydrogen (H_2_).[^19^, ^20^, ^21^] Activated carbons have been considered for hydrogen storage for decades,^[22]^ but the range of available adsorbents has increased substantially in recent years due to advances in the synthesis of stable porous framework materials.[^23^, ^24^, ^25^] High gravimetric capacities, exceeding 10 wt.% for some MOFs (e. g. MOF‐177),^[26]^ are achievable using H_2_ adsorption at low temperatures (e. g. 77 K) and moderate pressures, but ambient temperature capacities and volumetric densities remain rather low.

Aside from storage capacity, other properties are also important for practical applications, including cyclic stability, H_2_ sorption kinetics, and the enthalpy of absorption or adsorption. Some materials, for example, are highly stable, whereas others degrade during cycling, thus limiting the number of times a storage tank can be refilled to full capacity.^[27]^ Slow kinetics, meanwhile, prevent rapid recharging, and high enthalpies lead to more heat being generated during filling. This heat must be dissipated, so thermal management becomes more challenging.[^28^, ^29^] Other practical issues such as material cost and ease of production are also, of course, critical.^[27]^

The growing interest in hydrogen storage during the 1990s was unfortunately accompanied by publication of a number of examples of irreproducible data. Reproducibility, and the replication of results, is a cornerstone of science,^[30]^ but it has also been attracting increasing attention in a number of different disciplines due to the amount of irreproducible data present in the literature.[^31^, ^32^, ^33^] In 2019, the US National Academies of Sciences, Engineering and Medicine published a report on the issue,^[34]^ concluding that, despite some progress, more effort is required to help improve the reproducibility and replicability of science.^[35]^ We recently detailed cases of irreproducibility in hydrogen storage material research and discussed possible reasons behind the problems, including the effect of sample purity on the hydrogen sorption properties of materials and the sensitivity of hydrogen sorption measurements to error.^[36]^ A subsequent study by Sholl and co‐workers, in a related area, found that around 20 % of the published results they analysed, on CO_2_ adsorption by MOFs, are unlikely to be reliable.^[37]^

With regard to hydrogen sorption measurements, the potential errors are well understood,[^6^, ^38^] but they are difficult to quantify or control. Errors, both systematic and random, can become prohibitively large, for example, when making measurements on small samples; although a general sample size threshold cannot be defined because the amount of error depends on various factors, including the measurement technique, instrument, and material. It is nevertheless imperative that care is taken when performing hydrogen sorption measurements, particularly on small samples, and that researchers view any surprising results with healthy scepticism in light of the documented episodes of irreproducibility in the field.^[36]^

Clearly erroneous data are still being published and this continues to hinder progress. A range of experimental uptakes, for example, have been claimed for graphene‐based materials, but hydrogen sorption measurements performed by Talyzin and co‐workers on different graphene‐based materials showed that high pressure capacities at ambient temperature and 77 K scaled with surface area, in contrast to a number of previous reports.^[39]^ It is critical that claims of high hydrogen storage capacity are made on the basis of reliable, repeatable and reproducible results, rather than the scientific novelty of the material – the unique electronic properties of graphene,^[40]^ for instance, will not necessarily lead to increased hydrogen uptakes. Graphene‐based materials have also been involved in recent claims regarding the use of hydrogen spillover for hydrogen storage, an area of research that has been notably affected by reproducibility problems.[^21^, ^36^, ^41^, ^42^, ^43^] See, for example, the Comment by Klechikov and Talyzin.^[44]^

A number of approaches could help improve the situation.^[36]^ These include standardisation of measurement methods, definition of measurement or reporting guidelines, increased collaboration between both theoreticians and experimentalists, and different laboratories, in order to help corroborate research results, and increased use of various tests and cross‐checks to help determine the validity of each set of hydrogen sorption data. Publication of more independently‐validated data would also be welcome. Nevertheless, the most obvious and simple to resolve issue is the lack of accepted measurement and reporting guidelines, a situation this article therefore seeks to address. We begin by describing the main measurement techniques, before defining typical measurement procedures and discussing troubleshooting. We then provide reporting guidelines, defining the information that should be provided when publishing hydrogen sorption data.

## 2 Measurement Techniques

The two approaches discussed in this article are the manometric and gravimetric techniques; the former of which is also known, for historical reasons, as Sieverts’ method or the volumetric technique. Commercial instruments implementing both approaches are now widely available, but self‐built manometric systems, in particular, are also commonly used, as they can be readily constructed from off‐the‐shelf pressure and vacuum components. The main technical benefits of commercial manometric instruments include their full automation and specific design features, such as minimised internal dead volumes, optimal valve and pressure component selection, and sample cells appropriate for containing fine powders.

**Figure 1:**
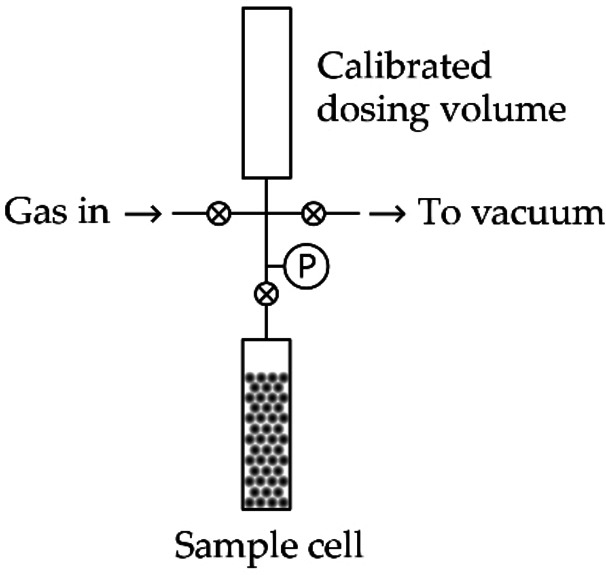
A schematic diagram of a basic manometric H_2_ sorption measurement system.^[6]^

A basic manometric instrument is shown in Figure 1. It consists of a calibrated dosing volume and a sample cell, separated by a valve and connected to a gas supply and vacuum pump. Using a dry, high vacuum system – such as a turbomolecular pump backed by a diaphragm (or membrane) pump – will provide high vacuum conditions (<10^−1^ Pa) and avoid contamination from backstreaming oil vapour.[^6^, ^38^] Manometric instruments must also be leak tight. All‐metal construction is typical for systems designed for use with hydrogen because elastomer o‐rings, for example, provide less robust seals than metal‐to‐metal gasket or cone fittings, and are therefore more susceptible to H_2_ leakage.

Due to the measurement principle – as described later – the overall accuracy of a manometric instrument depends mainly on its internal volume and the pressure measurement accuracy, although the temperature of the system must also be stable throughout a measurement. The temperature of the dosing volume and sample cell should therefore be controlled and measured accurately, with the amount of exposed, unthermostatted volume minimised, to reduce the susceptibility of measurements to room temperature fluctuations.^[38]^ Temperature measurement accuracy is ultimately limited by the accuracy of the sensors. Type K thermocouples, for example, have typical accuracies of ±1 K, while platinum resistance thermometers (PRTs) have higher accuracies of ±0.1 K. Sensor positioning, however, is also important. The highest achievable pressure measurement accuracy, meanwhile, using commercial transducers, is currently around 0.01 % of full scale, although such devices are expensive. A more common level of accuracy for hydrogen sorption instruments is 0.1 % (or ±0.05 %).

**Figure 2:**
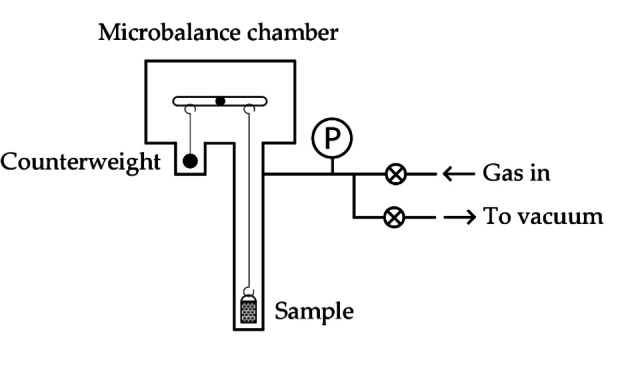
A schematic diagram of a basic gravimetric H_2_ sorption measurement system.^[6]^

A basic gravimetric instrument, meanwhile, is shown in Figure 2. It consists of a microbalance connected to a gas supply and vacuum pump system. In this case, weight changes measured by the microbalance are used to determine hydrogen uptake, in a similar manner to thermogravimetric analysis (TGA). In contrast to standard TGA instruments, however, a gravimetric gas sorption system must exhibit long term weight measurement stability, so that any weight change can be reliably attributed to hydrogen sorption or desorption, rather than microbalance drift. Commercial gravimetric gas sorption instruments typically have long term microbalance stabilities of ±1 μg. For TGA, this is less crucial because a measurement is usually quick; only the weight trace over the course of a few minutes or hours is needed, whereas gravimetric hydrogen sorption measurements often take much longer. Sample temperature must also be carefully controlled and measured, and pressure measured accurately to define the pressure of each isotherm point. The overall measurement accuracy of a gravimetric instrument therefore depends mainly on the accuracy and long term stability of the microbalance, the temperature stability, and pressure and temperature measurement accuracy.

## 3 Procedure

### 3.1 Manometric Technique

To begin a manometric measurement, a known quantity of sample is loaded into the cell, which is then attached to the instrument and sealed. The system is then evacuated, and the integrity of the pressure seals confirmed, by monitoring – for example – the pressure above the vacuum pump. Failure to reach the expected base vacuum indicates leakage, which must be avoided in order to make accurate manometric sorption measurements.

The sample must then be activated or degassed, a process that depends on material type (see below). Once activation is complete, the calibrated dosing volume is pressurised with H_2_ gas and the pressure measured. The separating valve is then opened to dose gas to the sample and the pressure (and temperature) monitored to assess the approach to equilibrium. Once equilibrium has been achieved, measuring temperature and pressure allows calculation of the absorbed or adsorbed quantity using a molar balance expression. This pressurisation and dosing process is then repeated until a full isotherm has been determined. Reversing the procedure – by successively decreasing the pressure in the dosing volume and opening the valve each time to remove H_2_ from the sample cell – allows determination of a desorption isotherm.

### 3.2 Gravimetric Technique

In the gravimetric case, the sample is loaded onto the microbalance pan (or holder), the chamber sealed, and the system evacuated. Integrity of the pressure seal must again be confirmed, and the sample degassed or activated. To begin an isotherm measurement, the sample is exposed to a pressure of H_2_ gas and the weight monitored as hydrogen sorption occurs. Once equilibrium has been achieved, in terms of sample weight, temperature and pressure, the measured weight is then used to calculate the absorbed or adsorbed quantity, by applying buoyancy corrections. The gas pressure is then increased further and the above process repeated, until a full isotherm has been determined. Again, reversing the procedure – by decreasing the pressure in steps and measuring the equilibrium weight at each point – allows determination of a desorption isotherm.

### 3.3 Activation and Degassing

As noted above, a sample must be fully activated or degassed prior to isotherm determination. The precise procedure or requirements depend on the material.

For metallic elements or compounds that form interstitial hydrides, activation is required to prepare the material for reversible hydrogen absorption. Two main issues must be addressed. Firstly, surface passivating layers, such as oxides, usually hinder the reaction of H_2_ with materials, and must therefore be reduced or cracked to allow hydrogen to enter the bulk of the sample. Secondly, materials often decrepitate upon hydrogen absorption, due to the lattice expansion associated with hydride phase formation.[^45^, ^46^, ^47^] Repeated hydrogen cycling then leads to further particle size reduction, before fully reversible hydrogen absorption is observed.^[47]^ When powder particles crack, fresh surfaces are exposed, and this further facilitates the hydrogen absorption process. For each material, an appropriate activation temperature, hydrogen pressure, and number of cycles must therefore be chosen, to ensure a sample is fully activated. Various examples can be found in the literature.[^11^, ^48^, ^49^, ^50^]

Complex hydrides, meanwhile, are usually synthesised in a hydrogenated state, so they do not require activation. However, they are typically air and moisture sensitive, so special care must be taken, for example, by handling samples in an inert environment,[^13^, ^51^] using a glovebox or Schlenk line.

Nanoporous materials, in contrast, only need to be degassed, although some are air and moisture sensitive and must be handled in an inert environment.^[21]^ Examples include MOFs with open metal sites and any high surface area material doped with small metal clusters^[52]^ that can easily oxidise. MOFs also usually have solvent molecules trapped in their pores, following synthesis, and these must be removed – a process also known as *activation*.[^21^, ^25^] MOFs, therefore, must be fully activated first to remove solvent, before then being degassed to remove any other pre‐adsorbed species.

IUPAC guidelines on physisorption measurement^[53]^ suggest the surface of a microporous material should be exposed to high vacuum, at pressures <1 Pa, for thorough degassing. Elevated temperatures are also usually required, depending on the thermal stability of the material. An appropriate degassing temperature for any new material can be estimated using TGA, to assess its thermal stability. More delicate materials, including most MOFs and organic polymers, may be restricted to lower temperatures, around 100 °C to 200 °C (373 K to 473 K), whereas more robust materials, such as zeolites and porous carbons, can often be degassed at 300 °C (573 K) or above. It is important, however, to avoid modifying or damaging the material during degassing. For hydrophilic materials, such as low silica zeolites, increasing the temperature in steps is sometimes necessary, because of their susceptibility to hydrothermal decomposition. Most of the residual water adsorbed in the pores is removed at a lower degassing temperature, before higher temperatures are applied to complete the process. Nevertheless, regardless of the exact details, ensuring a nanoporous material is fully degassed prior to a measurement is an essential step in accurately measuring H_2_ adsorption.

### 3.4 Calculating Hydrogen Uptake

Calculating the absorbed or adsorbed quantity at each isotherm point, using either the manometric or gravimetric technique, depends on the configuration of the apparatus. Manometric measurements, however, generally use a molar balance expression of the form,

(1) $$\Delta n=\frac{P_{i}V_{1}}{Z_{Pi,T}RT}-\frac{P_{f}\left(V_{1}+V_{2}\right)}{Z_{Pf,T}RT}$$

where *Δn* is the number of moles absorbed or adsorbed, *P_i_* and *P_f_* are the initial and final pressures, *V_1_* and *V_2_* are the dosing volume and dead volume of the sample cell, respectively, *R* is the universal gas constant, and *T* is temperature. *Z*_*Pi,T*_ and *Z*_*Pf,T*_ are the compressibility factors of H_2_ gas at the pressures *P_i_* and *P_f_*, and temperature, *T*. Note that *V_2_*, the dead volume of the sample cell, depends on the sample volume, as *V_2_* =*V_cell_*−*V_s_*, where *V_cell_* is the empty sample cell volume and *V_s_* is the skeletal volume of the sample. Either *V_2_* or *V_s_* must therefore be determined accurately. This typically involves either directly determining *V_2_* using He, or performing an independent He pycnometry experiment to measure the sample skeletal density, *ρ_s_*. For nanoporous materials, it is important not to perform a direct *V_2_* measurement at low temperatures, such as 77 K, as helium will adsorb in micropores under such conditions,^[54]^ leading to an error in *V_2_*.

**Figure 3:**
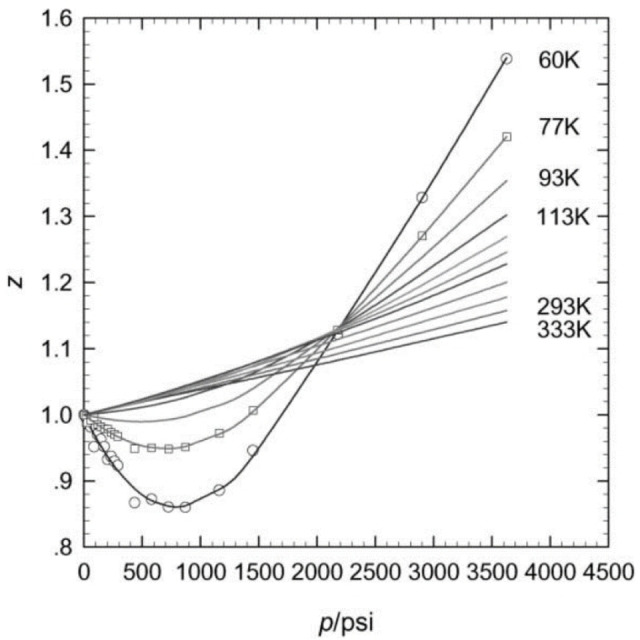
Compressibility factor, *Z*, of H_2_ plotted as a function of pressure, for various temperatures, in the range 60 K to 333 K, up to 25 MPa (1 MPa=145 psi). The points are experimental data and the lines are polynomial fits. Reproduced with permission from Ref. [56]. Copyright (2001) Elsevier.

Equation (1) applies to an entirely isothermal system, but it is important to note that the sample cell is often held at a different temperature to the dosing volume, which is usually maintained at near ambient temperature. In this case, real calculations must therefore account for the temperature difference.[^6^, ^38^, ^55^] This can have a particularly severe effect at low sample temperatures, such as 77 K, due to the large temperature gradient under these conditions and the significant differences in *Z*_*P,T*_ at near ambient and low temperatures. Accurate corrections also become much more important at higher measurement pressures because deviations in the compressibility factor of H_2_ at different temperatures increase significantly as a function of pressure, as shown in Figure 3.^[56]^

Gravimetric measurements, meanwhile, require buoyancy corrections, to account for differences when measuring weight in a fluid of differing density, depending on temperature and pressure. The total force on the microbalance, *f_tot_*, in a sorption experiment is given by,

(2) $$f_{tot}=f_{sorb}-f_{buoy}=m_{sorb}g-m_{s}g\left(\frac{\rho_{g}}{\rho_{s}}\right)$$

where *f_sorb_* and *f_buoy_* are the forces due to sorption and buoyancy, *m_sorb_* and *m_s_* are the masses of absorbed or adsorbed gas and the degassed (empty) sample, *ρ_g_* and *ρ_s_* are the densities of the bulk gas phase and the sample, respectively, and *g* is the acceleration due to gravity. If *ρ_g_* and *ρ_s_* are known, the amount of hydrogen absorbed or adsorbed can then be calculated from the microbalance signal, referenced to the degassed sample mass obtained under vacuum at the start of the experiment. Note that determining *ρ_s_* is the equivalent of determining *V_s_* for manometric measurements, as *ρ_s_* =*m_s_*/*V_s_*. Real calculations are, again, more complicated because other components are often present in the microbalance chamber and therefore contribute to the overall buoyancy effect, so this must be accounted for, together with temperature differences in different parts of the apparatus. Further details of the calculations required for both techniques can be found elsewhere.^[6]^

For H_2_ adsorption in nanoporous materials, it is important to note that, if the volume of the adsorbed phase is not accounted for, the calculated adsorbed quantity is the *excess adsorption*.[^57^, ^58^] Other definitions of adsorption, however, include *net*,^[59]^ *absolute*[^60^, ^61^] and *total*,^[62]^ each of which require different assumptions; although there is currently no consensus on the best choice.^[63]^ Practically, it depends on the information required. Net adsorption, for example, provides direct information on the benefit of filling a storage tank with adsorbent, while absolute adsorption is required for thermodynamic calculations, such as determining the isosteric enthalpy of adsorption.[^61^, ^63^, ^64^, ^65^] H_2_ adsorption isotherms for storage applications, however, are commonly reported as excess adsorption. The only assumption required to calculate excess is that the volume of the sample can accurately, or at least consistently, be determined using He pycnometry.^[54]^ This defines *V_2_* in Eq. (1) and *ρ_s_* in Eq. (2). Note that sample volume or density is also required for measurements on metal or complex hydrides. In this case, the expansion or density change of such compounds during hydrogen absorption must be considered, particularly if *V_2_* or *ρ_s_* are altered significantly as a result. The importance of this must be assessed on a case‐by‐case basis.

### 3.5 Capacity Definitions

The above describes the principles of calculating hydrogen uptake using either technique, but the hydrogen storage capacity of a material can be defined in different ways. As noted above, for example, gravimetric H_2_ adsorption capacities can be reported as net, excess, absolute or total.^[19]^ Alternatively, volumetric capacity, which is important for practical applications,[^27^, ^66^, ^67^] can be reported. This depends on how the material volume or density is defined, regardless of material type. Using the bulk density of a powder bed, for example, which is realistic for practical hydrogen storage systems, will result in a lower calculated volumetric capacity than the value obtained using the crystal density of a single particle.^[27]^ For comparison, consistent definitions must be used, but inappropriate choices can also result in falsely inflated capacity values or unrealistic claims of a material's performance potential. From a practical perspective, the possibility of calculating different usable or working capacities,[^68^, ^69^] depending on the chosen storage and delivery pressures, adds further complication.^[21]^

## 4 Sample Characterisation

Problems have occurred in hydrogen storage material research for a number of reasons. Measurement issues are one aspect, but difficulties with accurately characterising materials and assessing material purity are another. Before discussing the troubleshooting of hydrogen sorption measurements in more detail, we will therefore briefly discuss sample characterisation.

Materials studied for hydrogen storage are synthesised using a range of different methods, the details of which are beyond the scope of this article. It is important to note, however, that most synthesis routes can result in the presence of impurities in samples that may significantly alter the interaction of H_2_ with the material. Examples of impurities include different chemical species or minority phases, such as precipitates of differing structure and stoichiometry or amorphous regions in an otherwise crystalline material. Identifying the presence of such impurities relies on thoroughly characterising each material, which can be challenging, particularly for nanostructured composites synthesised using multistep processes. Global characterisation techniques, such as diffraction or chemical analysis, for example, may miss defects and small impurities, while local methods, such as high‐resolution electron microscopy, may focus only on an atypical part of the sample. Purely crystalline materials can be characterised using X‐ray or neutron powder diffraction, but careful attention must be paid to data collection and analysis, according to established guidelines.^[70]^ Powder diffraction also has detection limits for crystalline minority phases and may miss amorphous phases entirely.^[71]^ Gas adsorption analysis, meanwhile, which is a complementary technique for determining the surface area, pore size distribution, and pore volume of porous materials, must be applied with care, as it can be subject to both measurement error and misinterpretation.[^53^, ^72^, ^73^, ^74^, ^75^]

Regardless of the details of each characterisation technique, however, it is important to apply an appropriate set of methods to each material, and to avoid common pitfalls, as discussed, for example, by Weidenthaler.^[74]^ Carefully characterising a sample prior to H_2_ exposure and after a sorption cycle is the key to independently reproducing and comparing experiments performed by other researchers.

## 5 Troubleshooting

Following sample synthesis and characterisation, various aspects of the measurement of hydrogen sorption must be considered. This section covers key areas of troubleshooting, grouped into those relating to instrument set‐up (gas purity, leaks, blank scans and system validation); measurement strategy (sample size, sample pretreatment and activation, temperature control and measurement, the chosen number of isotherm points, and equilibration times); aspects of data processing (real gas behaviour); and assessing problems with hydrogen sorption data (isotherm shape, repeatability, and the possible occurrence of catalytic reactions between H_2_ and the sample).

### 5.1 Gas Purity

Most impurities present in H_2_ can interfere with hydrogen sorption measurements. Research grade H_2_ (>99.999 % purity) must therefore be used, preferably with further purification near to the instrument.^[76]^ Moisture contamination, particularly for gravimetric measurements, must be avoided. Commercial gas purifiers are available, but liquid N_2_ traps are effective for removing impurities, by freezing out any vapour phase species.^[38]^ Sufficient H_2_ purity can be confirmed by making measurements on well‐understood materials, as discussed later, providing they are as sensitive to impurities as the studied samples. In severe cases, gas purity can be assessed using mass spectrometry, which should detect specific contaminants such as H_2_O and hydrocarbons. Appropriate action can then be taken, for example, by changing the gas supply or using purification specific to the identified contaminants. Test measurements can then be repeated to confirm the success of such measures.

### 5.2 Leakage

Leakage, particularly in the manometric technique, can lead to false measurement of absorption or adsorption.^[77]^ If pressure decreases due to outboard leakage, for example, *P_f_* in Eq. (1) will be lowered, falsely increasing the calculated value of *Δn*. In this case, the amount of calculated hydrogen uptake will increase with the amount of time allowed for equilibration. However, other scenarios, including internal leakage through valves, are also possible, each of which will lead to erroneous calculations of the hydrogen content of a material. Manometric instruments must therefore be checked for leaks prior to making a measurement. Blank scans, which are addressed next, can be used to identify problems. The amount of error due to leakage will tend to increase with pressure, leading to curvature in blank scan or test isotherm data at higher pressure. Curvature towards either higher or lower hydrogen content can occur, depending on the direction of leakage. Increasing the measurement pressure further will therefore help identify leakage as a problem.

Potential causes of leakage include insufficient tightening of pressure fittings, contaminated valves, damaged pressure seals and valve seats, and hydrogen permeation through the walls of sample cells at elevated temperature (see, for example, Sheppard et al.).^[78]^ Valves can become contaminated by fine powder, so samples must be secured in the sample cell or holder, to prevent contamination. Quartz wool can provide an effective barrier, to prevent powder particles contaminating the rest of the system, but valves can also be protected by commercially available sintered filter gaskets. Damaged or contaminated components may need to be replaced. Hydrogen permeation through sample cell walls, meanwhile, may indicate that measurements are being made beyond the limits of operation of the instrument. This will depend on the choice of construction material. Hydrogen permeates through different grades of stainless steel (SS), for example, at significantly different rates,[^79^, ^80^] although 316 L SS is standard for high pressure H_2_ systems and is resistant to permeation up to relatively high temperatures.^[78]^ Nevertheless, the source of any leakage must be investigated thoroughly. Removal and cleaning of components is sometimes necessary, prior to re‐testing a system and then using a process of elimination to achieve leak‐free operation.

### 5.3 Blank Scans

All H_2_ sorption isotherm measurements are referenced to sample volume, whether from an estimated or measured material density, or a directly determined dead volume (see the Procedure section). Measuring isotherms with helium, which should not appreciably absorb or adsorb at ambient temperature or above, can thus be used to test and demonstrate accurate instrument calibration, prior to making a H_2_ sorption measurement. Such blank scans, together with empty sample cell or sample holder runs with H_2_, can be used to ensure an instrument is free of leaks, in the manometric case, and that any necessary volume, weight, pressure and temperature measurement calibrations are correct, regardless of technique. Unexpected H_2_ sorption results can also be checked by removing the sample and subsequently performing such scans at the measurement temperature, to confirm the instrument is operating correctly. The key point is that empty sample cell isotherms measured with H_2_ should result in no calculated sorption for all pressures, within the expected uncertainty based on the instrument specifications.^[38]^ Any significant deviation indicates problems with instrument performance and calibration, or aspects of data processing, such as the choice of equation of state (EOS), as discussed later, or the corrections required to account for temperature differences in different parts of the apparatus.

### 5.4 System Validation

In addition to performing blank scans, system operation should also be validated by performing measurements on materials for which isotherm shape and hydrogen storage capacities are well established. Unfortunately, no certified reference materials are available for hydrogen storage, but well‐understood materials, such as Pd, LaNi_5_ and commercial activated carbons, for example, can be used as proxies. Measuring H_2_ sorption by a material similar to that being studied, and at the same temperature and pressure, is an important way of demonstrating correct calibration and operation of equipment. Such measurements should also be made with a comparable amount of sample. Note that making apparently accurate measurements of hydrogen absorption by Pd or LaNi_5_, at ambient temperature or above, will not guarantee that an instrument can measure accurate high pressure H_2_ adsorption isotherms at 77 K on a nanoporous material.[^38^, ^55^, ^81^] This is because the densities of these materials differ considerably, which affects measurement sensitivity to sample density errors that are greater for low density porous materials.^[81]^ Near ambient temperature measurements are also not affected by the severe temperature gradients present when making measurements at low sample temperatures – as discussed in the Procedure section. The importance of this point cannot be overstated.

### 5.5 Sample Size

**Figure 4:**
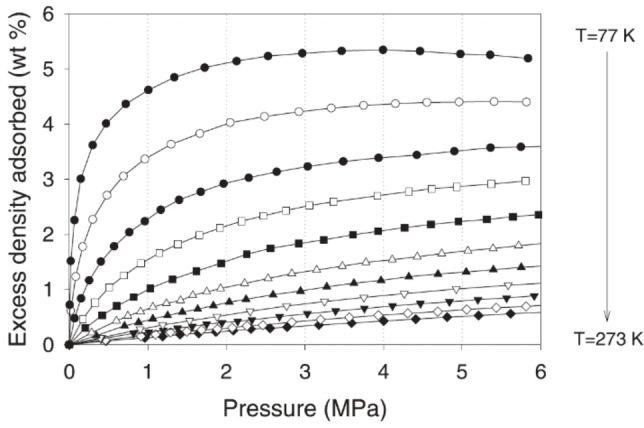
Excess H_2_ adsorption isotherms for a commercial activated carbon, AX‐21,^[82]^ measured in the range 77 K to 273 K. Reproduced with permission from Ref. [83]. Copyright (2004) Springer Nature.

When making H_2_ sorption measurements, sample size choice is critical. The main difficulty encountered is using too small a sample. The lower threshold for any material depends on the instrument specifications and performance, although temperature may also play a role because uptakes – for adsorbents, in particular – may be very low at certain temperatures. For example, the amount of H_2_ physisorbed in nanoporous materials, at any particular pressure, decreases with increasing temperature, so uptakes are typically low at ambient temperature or above. More sample may therefore be required for higher temperature measurements on nanoporous materials, in any given instrument. To illustrate the change in uptake with temperature, Figure 4 shows excess H_2_ adsorption isotherms measured for an activated carbon in the temperature range 77 K to 273 K. Only small amounts of uptake are observed at the highest temperatures, even at 6 MPa (60 bar). Note that around 20 grams of sample was used for these measurements.^[82]^ This is a large quantity compared to those typically used in research reports. Only tens of milligrams of some new materials may be available, although it is more common to use sample sizes in the range from 100 mg to 1 or 2 g for hydrogen sorption measurements.

The sensitivity of a manometric measurement mainly depends on the total internal volume of the system, the ratio of the dosing and sample cell volumes, and pressure measurement accuracy.[^38^, ^81^, ^84^, ^85^] Each instrument therefore has a minimum amount of hydrogen uptake it can reliably detect, which can be calculated to a first approximation from the instrument specifications. The sensitivity of a gravimetric measurement, meanwhile, depends mainly on the accuracy and stability of the microbalance;^[38]^ although poor pressure and sample temperature measurement accuracy, together with poor temperature stability, will also limit the ability of an instrument to reliably detect small hydrogen uptakes. For both measurement types, blank scans – as discussed above – can help determine the background signal, which should combine all the effects in a given instrument, including both random and systematic errors.

**Figure 5:**
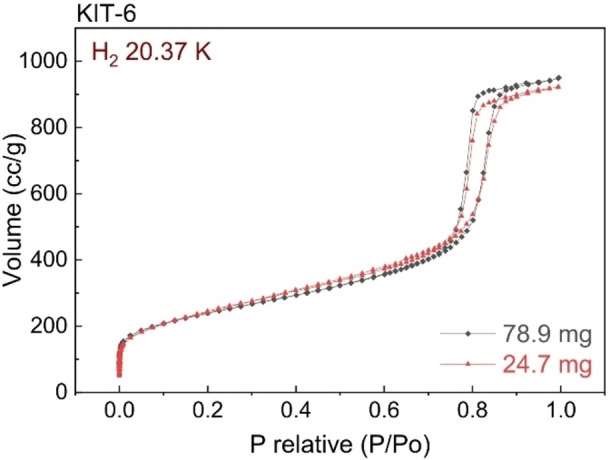
H_2_ adsorption isotherms measured manometrically, for the mesoporous silica KIT‐6 at 20.37 K, illustrating good agreement between data determined using two different sample masses.^[86]^

Information regarding the background signal of an instrument, and the minimum amount of hydrogen uptake it can reliably detect, should allow a realistic assessment of the minimum sample size. To identify problems, however, measurements can be repeated using different size samples – see, for example, Figure 5.^[86]^ This should crucially indicate issues with sample size because the normalised hydrogen storage capacity of a material, expressed, for example, in wt.%, should be independent of the quantity of sample used. Note that most instruments will also have an upper sample size limit, which may be lower than the quantity of material that can be simply packed into the sample holder or cell.^[38]^ This possibility should therefore be considered, if sample size‐dependent results are obtained using relatively large samples.

### 5.6 Sample Pretreatment and Activation

Every H_2_ sorption measurement must be preceded by adequate pretreatment of the sample. Nanoporous materials require degassing, while metal hydrides must be activated,[^45^, ^49^] as discussed in the Procedure section. Varying the conditions – for example, degassing times and temperatures for adsorbents – and repeating isotherm measurements can be used to confirm that results are not significantly affected by different pretreatments. Measuring repeat isotherms on metal hydrides, meanwhile, will help confirm a sample has been fully activated, as hydrogen absorption and desorption should be repeatable following activation, even if the material may eventually degrade under long term cycling tests.

### 5.7 Temperature Control and Measurement

Sample temperature must be carefully controlled during a measurement. Ambient temperature fluctuations may be an issue, but gas expansion through orifices and the reaction of H_2_ with materials can cause temperature excursions, which can be mistakenly interpreted as sorption or desorption.^[77]^ It is therefore necessary to ensure that the sample temperature and gas temperature (both in the dosing volume and sample cell in the manometric case) have stabilised and reached equilibrium before calculating hydrogen uptake. Problems can be particularly severe with large dosing steps because thermal effects, due to gas expansion, increase with increasing pressure differential and more heat may be generated during the sorption process, with larger concentration changes.

Temperature changes will affect the equilibrium amount of sorption in most materials, due to the thermodynamics of the process; so even small variations in temperature during an isotherm measurement will affect the quality of data, regardless of technique. However, in the manometric case, calculations of the uptake will also be affected, adding to the total accumulated error. Heat generated during absorption or adsorption, meanwhile, will result in sample temperature increases. At each step of an isotherm, the sample temperature must therefore be monitored, to ensure that thermal equilibrium has been achieved, before a measurement is made.^[84]^ Otherwise, an isotherm will be determined under non‐equilibrium conditions and will therefore be inaccurate.

Another difficulty with temperature control can arise when making manometric H_2_ adsorption measurements at 77 K using liquid N_2_ (LN_2_) as a cryogen. In this case, it is important to ensure that the LN_2_ level does not change significantly through the course of a measurement, as this will alter the temperature gradient in the apparatus.^[73]^ The main consideration is boil‐off, which will change the LN_2_ level as a function of time. Volume calibrations must also be performed under the same conditions. Otherwise, any difference in the conditions used for calibration and measurement will contribute to measurement error. The sensitivity of the calculated H_2_ uptake to the temperature gradient, in this case, is such that this should be one of the first possibilities considered when unexpected or surprising results are obtained for H_2_ adsorption at 77 K.

### 5.8 Number of Isotherm Points

**Figure 6:**
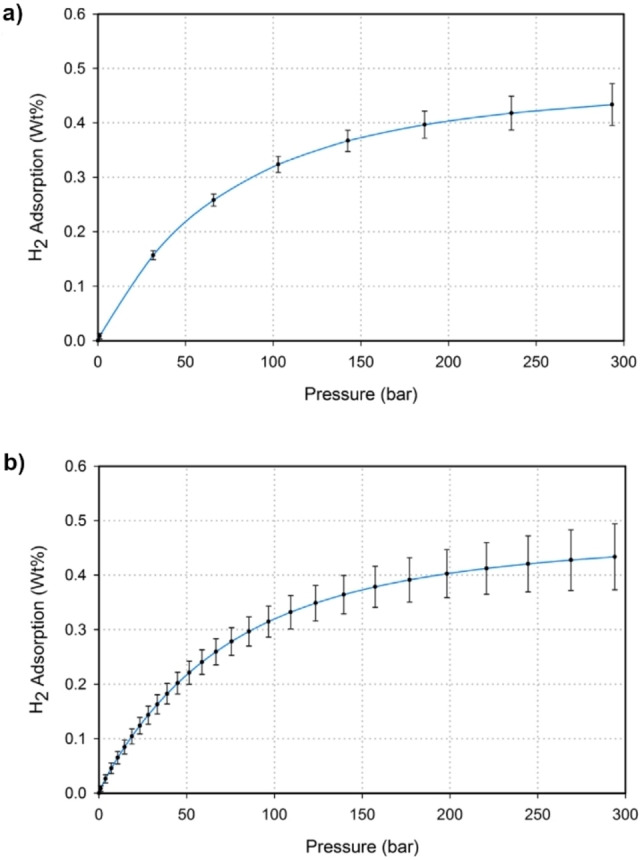
H_2_ adsorption isotherms measured manometrically, at 300 K on 1.37 g of Takeda 4 A carbon, illustrating the difference in uncertainty depending on the number of isotherm points, using a) 8 steps and b) 27 steps. Reproduced with permission from Ref. [85]. Copyright (2014) Elsevier.

The overall accuracy of a manometric sorption measurement can be affected by the number of equilibrium points used for isotherm determination, due to error or uncertainty accumulation. This is illustrated in Figure 6, which shows the increased uncertainty in H_2_ adsorption isotherms measured using more points, for the same material, in the same instrument. It is therefore important not to use too many pressure steps, as this will unnecessarily increase any error and uncertainty in final calculated uptakes.^[85]^ It is only necessary to measure enough points to obtain the required amount of information from an isotherm. This is complicated in some materials, however, because metal hydrides, for example, can be affected by the so‐called *large aliquot effect*, in which different size pressure steps in manometric measurements result in differing equilibrium pressures for any given hydrogen‐to‐metal ratio.[^38^, ^87^, ^88^] Problems can be identified by measuring repeat isotherms with a different number of isotherm points, to determine the effects of either accumulative error – which will affect both adsorption and absorption – or the large aliquot effect in metal hydrides.

### 5.9 Equilibration Times

**Figure 7:**
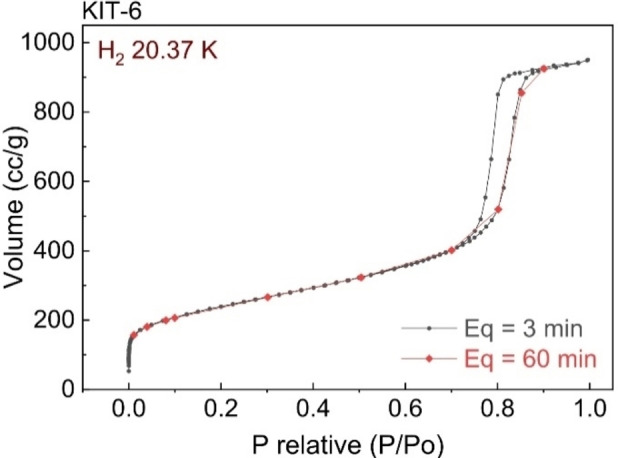
H_2_ adsorption isotherms measured manometrically, for the mesoporous silica KIT‐6 at 20.37 K, illustrating good agreement between data determined using two different equilibration times.^[86]^

H_2_ sorption isotherms should describe equilibrium behaviour, but the kinetics of H_2_ sorption can vary dramatically between different materials, at different temperatures and pressures. Sufficient time must therefore be allowed for equilibration at each isotherm point, and the method and criteria used to determine equilibrium should be considered carefully. H_2_ adsorption by nanoporous materials generally requires only minutes at each isotherm point,[^89^, ^90^] although in materials possessing particularly small pores more time may be needed, due to slower diffusion rates.^[91]^ Metal or complex hydrides, meanwhile, can take hours or even days to reach equilibrium.[^48^, ^51^, ^88^, ^92^, ^93^] Time‐dependent data can be checked at each isotherm point, after completing a measurement, to ensure equilibrium has been achieved. Repeat measurements can again be used to assess problems, by lengthening the equilibration times or tightening up the equilibrium criteria, if they can be set in instrument control software, for example, in commercial systems – see Figure 7.^[86]^ Non‐equilibrium conditions can often be detected from the shape of isotherms. If the points at the start of the plateau region of a metal hydride isotherm, for example, are higher in pressure than those at higher hydrogen‐to‐metal ratios, this indicates that equilibrium has not been achieved. In this case, lengthening the equilibration times will result in differences in the subsequent isotherms, thus confirming likely problems with this issue, in the absence of leakage.

### 5.10 Real Gas Behaviour

**Figure 8:**
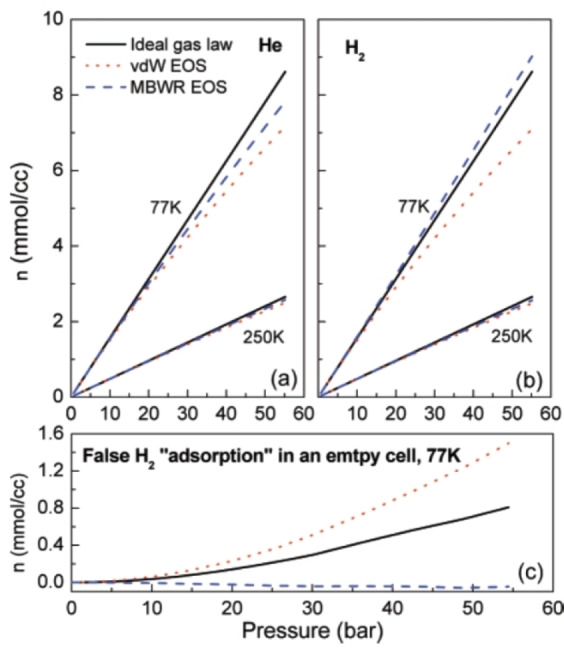
Plots of the molar density, *n*, of (a) He and (b) H_2_, as a function of pressure at 77 K and 250 K, and (c) the false measurement of H_2_ adsorption in a manometric instrument, depending on the chosen equation of state. Reproduced with permission from Ref. [94]. Copyright (2007) American Chemical Society.

All high pressure H_2_ sorption measurements require the real gas behaviour of H_2_ to be described accurately. Using the ideal gas law, for example, at above ambient pressure will therefore result in errors.^[94]^ An accurate EOS should thus be used to calculate *Z*_*Pi,T*_ and *Z*_*Pf,T*_ in Eq. (1) and *ρ_g_* in Eq. (2), and hence to accurately calculate hydrogen uptake. Figure 8 illustrates the false measurement of adsorption that can occur when using the ideal gas law for manometric measurements at 77 K.^[94]^ The current state‐of‐the‐art EOS for H_2_ is the Leachman et al.^[95]^ expression, as implemented in the NIST (National Institute of Standards and Technology) REFPROP database,^[96]^ although other accurate EOSs are available^[97]^ – see Broom^[6]^ for further details.

The most obvious test, from a troubleshooting perspective, is to recalculate hydrogen uptake using different EOSs. The significance of any differences will depend on temperature and pressure, due to variations in H_2_ compressibility (i. e. non‐ideal behaviour) under different conditions, and this should be evident from testing the effects of using different EOSs. Measurements made at moderate pressures, up to a few bar (<1 MPa), and at ambient temperature or above, may not be affected significantly, but high pressure measurements – at 10 MPa or higher, for example – at low temperatures, such as 77 K, are likely to be particularly sensitive to EOS choice.^[94]^ Figure 3 shows a plot of *Z* as a function of pressure at different temperatures,^[56]^ illustrating the importance of the conditions on the deviation from ideal gas behaviour (i. e. *Z*=1). As noted earlier, inappropriate EOS choice is one reason why blank scans may not show essentially zero uptake for all pressures at the measurement temperature (see Figure 8), so this is another test worth trying.

### 5.11 Isotherm Shape

Isotherm shape is important because different types of H_2_‐solid interaction show notably different behaviour at different temperatures. Problems can often be identified, therefore, by assessing the shape of either individual isotherms or groups of isotherms measured at different temperatures.

Generalisations are difficult, but pure H_2_ physisorption by nanoporous materials at 77 K, for example, is typically characterised by Type I behaviour.^[53]^ In this case, the absolute adsorbed quantity increases significantly at lower pressures, before reaching a plateau, as it saturates at higher pressure. Excess adsorption, meanwhile, tends to peak at elevated pressures and then decreases as pressure increases further.[^19^, ^65^, ^82^, ^83^, ^94^] Example excess H_2_ adsorption isotherms for a range of temperatures are shown in Figure 4. A peak in the excess adsorption can be seen at around 4 MPa in the 77 K data.

Hysteresis is also not typically observed for H_2_ physisorption at low but supercritical temperatures, such as 77 K. At subcritical temperatures, it may be observed for mesoporous materials, as shown in Figures 5 and 7, due to the effects of capillary condensation, although such measurements are not common for hydrogen storage applications. At higher temperatures, however, hysteresis is characteristic of chemisorption. In rigid microporous materials, the presence of hysteresis in measured isotherms may therefore indicate gaseous impurity adsorption or other problems, such as kinetic limitations in small pore materials.^[91]^ Inaccurate volume calibrations in manometric instruments can also lead to systematic error accumulation through the course of an adsorption and desorption measurement. This will produce apparent hysteresis between the adsorption and desorption isotherms. Hysteresis observed during H_2_ adsorption measurement, at 77 K or above, should therefore be investigated with extensive validation measurements, to eliminate any of the above problems as potential reasons for measurement artefacts rather than real effects.

**Figure 9:**
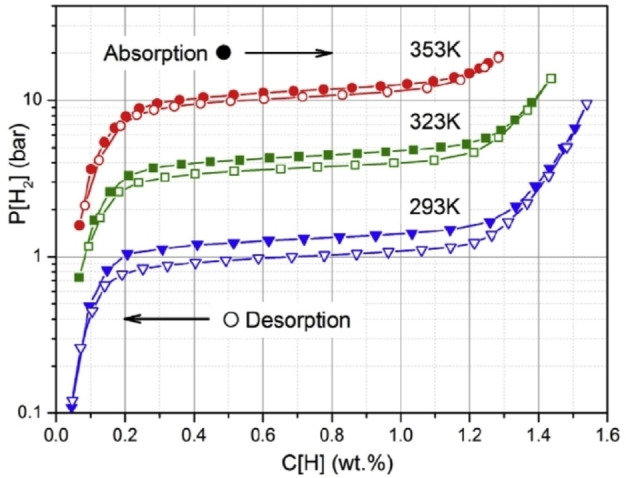
Hydrogen absorption and desorption isotherms for an AB_2_ alloy (Ti_0.15_Zr_0.85_La_0.03_Ni_1.2_Mn_0.7_V_0.12_Fe_0.12_), measured in the range 293 K to 353 K, illustrating behavior typical for interstitial hydrides, including an increasing plateau pressure with increasing temperature and hysteresis between the absorption and desorption isotherms, particularly at the lower temperature. Reproduced with permission from Ref. [99]. Copyright (2019) Elsevier.

In contrast to H_2_ physisorption by nanoporous materials, hysteresis between absorption and desorption is usually expected for metal hydrides.^[98]^ In this case, absorption occurs at higher pressures than desorption – see, for example, Figure 9.^[99]^ When both hydrogen absorption and desorption have been measured accurately, the hysteresis loop should close, at a finite pressure. If not, this indicates measurement problems, such as kinetic limitations or error accumulation. Desorption isotherms are also unlikely to cross the respective absorption isotherms, which is also the case for H_2_ adsorption. Such behaviour is therefore, again, indicative of measurement issues. With regard to isotherm shape, sloping plateaus, for interstitial metal hydrides, such as AB_5_ and AB_2_ compounds, are possible due to compositional gradients,^[100]^ but they are not generally expected for complex hydrides.^[88]^ It must therefore be ensured that observed behaviour is consistent with the physical or chemical nature of the host compound. If not, this indicates that measurements have not been performed accurately.

### 5.12 Repeatability

To help with troubleshooting, more than one consecutive isotherm can be determined to demonstrate the repeatability of a measurement. As well as indicating the lack of measurement problems – such as instrument calibration issues or the presence of impurities in the gas supply – reversible and repeatable H_2_ sorption should also demonstrate the absence of appreciable degradation of the sample, a critical requirement for practical applications. Confirming the repeatability of a measurement is a crucial precursor to subsequently ensuring the reproducibility or replicability of a result.

### 5.13 Catalytic Reactions

One final point to note is that, under certain conditions, catalytic reactions between H_2_ and the sample are possible, which can result in the formation of water,[^101^, ^102^, ^103^] C−H bonds,[^103^, ^104^, ^105^] surface hydroxyl groups,^[103]^ or hydrocarbons.^[106]^ This can occur, for example, in materials containing catalytic metals or surface oxides,[^101^, ^102^, ^103^] or when residual contaminants from the synthesis process may be present.^[106]^ It is also possible at ambient temperature – extensive literature exists on both water[^107^, ^108^, ^109^, ^110^, ^111^] and hydrocarbon^[112]^ formation under ambient conditions, in the presence of both H_2_ gas and metal catalysts (the discovery, by Döbereiner in 1823, of water formation over platinum under ambient conditions even gave rise to the term *catalysis*).[^110^, ^113^]

Such catalytic reactions will result in H_2_ consumption, and lead to a false measure of hydrogen sorption and potential overestimation of the reversible hydrogen storage capacity of a material. If this occurs, isotherm measurements are unlikely to be repeatable over multiple cycles, and so repeatability tests, as described above, can be used to check for such behaviour. It is sometimes necessary to pretreat a sample with H_2_, prior to degassing at an elevated temperature, in order to help first reduce oxide layers in doped samples and then subsequently remove the water.^[52]^ This will help prevent water formation during the subsequent hydrogen sorption measurements. Note that this is specific to the presence of oxidised metal particles, and that differing pretreatment procedures have been reported in the literature.[^52^, ^103^] Each sample must therefore be assessed on a case‐by‐case basis.

### 5.14 Summary

**Figure 10:**
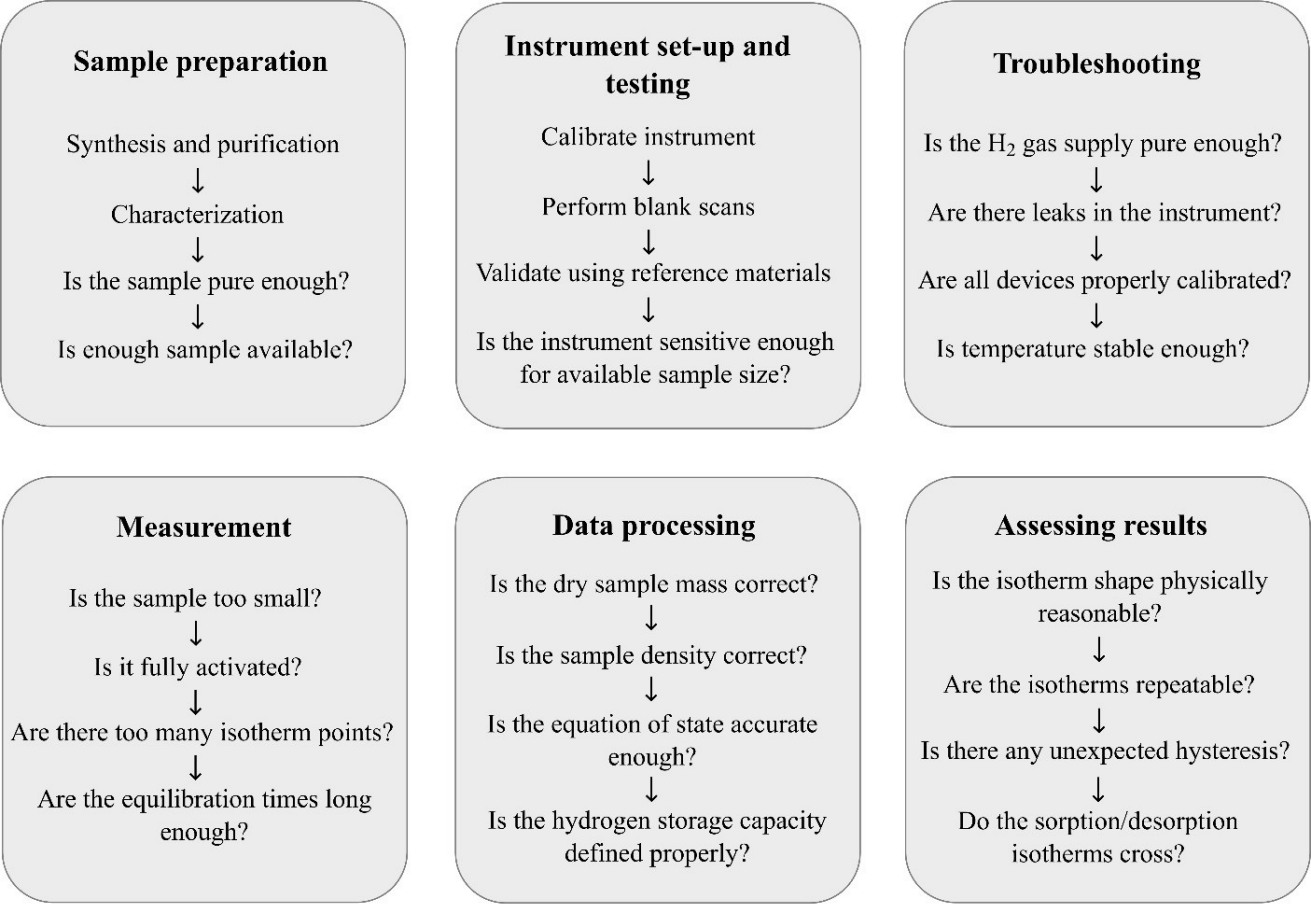
Six key stages of characterising the hydrogen storage properties of materials and troubleshooting problematic data.

To conclude this section, Figure 10 summarises the main stages of accurately characterising the hydrogen storage properties of materials and troubleshooting problematic data, in order to help increase the likely reproducibility of results.

## 6 Reporting Guidelines

The previous section covered key aspects of troubleshooting hydrogen sorption measurements and identifying potential sources of error. When reporting hydrogen sorption results, however, a minimum set of information should be provided. We now define the main points.

### 6.1 Instrument Specifications

Key details of the instrument or instruments used for H_2_ sorption measurement should be provided in any report. If commercial apparatus is used it may be sufficient to simply state the make and model used, but further information should ideally be provided. For manometric instruments, this includes the internal volume of the apparatus, pressure measurement accuracy, temperature stability and measurement accuracy, and the base vacuum of the system. Reporting such detail is particularly important when using self‐built systems. For gravimetric instruments, the balance resolution and long‐term stability should be stated, together with the method and accuracy of sample temperature control, pressure measurement accuracy, and the base vacuum of the system. If the above information is provided, an order of magnitude calculation of the detectable amount of hydrogen sorption should be possible. Alternatively, a minimum measurable H_2_ quantity can be stated.

### 6.2 Gas Purity

Gas purity should be clearly stated in any report. This can include the original purity quoted by the supplier, but any other purification should be described.^[76]^ This is important because it is possible that additional impurities are present in commercial gases, regardless of the supplier's specifications.^[114]^

### 6.3 Blank Scans

Blank scans, including those made with He or with H_2_ without a sample present, can be included as supplementary information to demonstrate that such tests were successful.

### 6.4 System Validation

Any validation measurements should be described, including H_2_ sorption isotherms measured on materials similar to those reported in the study. If the uncertainty in any measurement can be calculated, as a result of such validation tests, this should be included. The more information provided, the better.

### 6.5 Sample Size

As sample size choice is critical, the sample mass should be reported. For hydrogen uptake calculations, it is important to use the degassed sample mass. The method used to determine this should therefore be described. The sample density used for any uptake calculations should also be stated, together with the method used to determine either the sample density or volume.

### 6.6 Sample Pretreatment and Activation

The method used to pretreat or activate the sample must be clearly described. For metal hydrides, this should include a description of the activation procedure, such as the applied pressure and sample temperature, and the number of cycles used to achieve reversible absorption and desorption. For nanoporous materials, it should include the degassing temperature, vacuum level, and the amount of time used. Ideally, the method used to confirm that the sample has been fully degassed should be stated. This may include gravimetric degassing curves,^[68]^ or the fact that extending the degassing time, in the manometric case, did not alter the hydrogen uptake exhibited by the sample.

### 6.7 Temperature Control and Measurement

The stability of the sample temperature should be stated in reports. Ideally, the temperature control method should also be described, as this may dictate the achievable level of temperature control. For example, using LN_2_ should allow a stable sample temperature of 77 K to be achieved, whereas temperature stability when using a cryostat, furnace, or waterbath will depend on the control specifications.

### 6.8 Equilibration Times

The method and criteria for determining that equilibrium has been achieved at each isotherm step should be explicitly stated in reports. This may include fixed equilibration times, data fitting methods, or relevant weight or pressure change thresholds, depending on the technique.

### 6.9 Real Gas Behaviour

The EOS used should be stated and details provided in every report. This may include the software used to calculate the gas density, or H_2_ compressibility factors, as a function of temperature and pressure.

### 6.10 Capacity Definitions

The choice and definition of storage capacity should be stated in reports and any comparisons with previously published data made between directly comparable values. The method used to determine or define the material density, for volumetric capacity calculations, for example, should also be described.

### 6.11 Repeat Measurements

Repeat isotherms should be presented to demonstrate the repeatability of H_2_ sorption measurements.

### 6.12 Provide Sufficient Detail

The key point with regard to reporting experimental work is to provide sufficient detail to allow the replication of research results by other, independent researchers. Furthermore, if important details, such as sample mass, gas purity, and instrument specifications, are omitted from manuscripts, it is difficult – if not impossible – for editors or reviewers to assess the likely veracity of any hydrogen sorption data submitted for publication. As Stark^[115]^ pointed out, more generally, if a reviewer is given insufficient information about what was done, they will be unable to check it. Similarly, the 2019 US National Academies report on reproducibility and replicability^[35]^ stated that authors should include “a clear description of all methods, instruments, materials, procedures, measurements, and other variables involved in the study”. Any additional details required to replicate or assess reported hydrogen sorption measurements should therefore be provided.

## 7 Summary and Outlook

This article has described the manometric and gravimetric techniques for measuring hydrogen sorption, typical measurement procedures in each case, and the basic principles of calculating hydrogen uptake from measured values of weight, temperature, and pressure. Various aspects of troubleshooting problematic hydrogen sorption data and validating instrument operation have also been addressed, and reporting guidelines presented.

Previous interlaboratory exercises, performed on both nanoporous carbons^[116]^ and MgH_2_,^[117]^ have demonstrated the difficulties that can be encountered when trying to achieve reproducibility between results obtained in different laboratories.^[36]^ Recent efforts by Hurst and co‐workers, on nanoporous carbons, have achieved more consistent results,[^118^, ^119^] but the disparity observed in the earlier exercises[^116^, ^117^] does not inspire confidence in the likely veracity of all independently determined results published in the existing literature.

Reference materials, with known properties, are invaluable for validating measurement methods, but no such materials are currently available for either H_2_ adsorption or absorption. A CO_2_ adsorption isotherm for a NIST reference zeolite (RM 8852) was published in 2018,^[120]^ following an international interlaboratory study, and CH_4_ adsorption data for another material (NIST RM 8850) were reported last year.^[121]^ Similar H_2_ adsorption data, however – for a certified reference material – are not yet available, although an upcoming EURAMET project, MefHySto, will seek to address this issue.^[122]^ For metal hydrides, the situation is better because materials such as LaNi_5_ and Pd, which are widely available from chemical suppliers, exhibit relatively well understood hydrogen absorption behaviour; so these can be used to check instrument performance and calibration. Measurement error and uncertainty, however, depends on material density, and the problems identified during the 2013 MgH_2_ interlaboratory study^[117]^ show that care must be taken when making measurements on lower density metal hydride samples.

The pure single‐phase materials mentioned above are usually available in relatively large quantities. In contrast, research reports often feature novel nanomaterials, available only in very small amounts – sometimes just tens of milligrams. When this is the case, measurement accuracy becomes even more critical. Furthermore, the structure, composition and purity of such materials can be difficult to determine in detail and standards are lacking,^[123]^ thus rendering it difficult to know the exact nature of potentially complex materials. These factors are likely to have compounded the problems described in the Introduction to this article. Synthesis of the materials themselves may not be robustly reproducible, so it is perhaps unsurprising that H_2_ sorption results on such materials are also difficult to reproduce. This does not lessen the argument for trying to improve the reproducibility of H_2_ sorption results. It just emphasises the importance of improving reproducibility more generally.

In a recent Editorial, Sholl^[124]^ described five ways to help improve reproducibility, two of which are essentially covered by this article – showing validation data from tests on known materials and reporting observational details. The others include showing error bars on data, tabulating data in supplementary information, and providing input data and version information for computational results. Both the inclusion of error bars and data tabulation are relevant to experimental hydrogen storage material research. We have not included these in our reporting guidelines – the inclusion of error bars on hydrogen sorption data, in particular, is currently rather rare – but we encourage authors to consider these important additional points. There seems to be no good reason, for example, given current publication practices, for not providing tabulated data in supplementary information. The recent introduction by Evans et al.^[125]^ of a standardised Adsorption Information File (AIF), analogous to the Crystallographic Information File (CIF) used for structural data, will help further, for H_2_ adsorption results on nanoporous adsorbents.

Whether introducing reporting guidelines, or other such measures, will be enough to solve the problems in hydrogen storage material research is probably a moot point. However, more certainly needs to be done, as publication of irreproducible or erroneous data can be costly, due to the effort spent on attempted replication and the grants awarded as a direct consequence of the publication of headline‐grabbing, but ultimately irreproducible, results. Any strategies that can be implemented more generally to reduce this cost would be welcome, but we hope the above measurement and reporting guidelines at least represent a helpful step in the right direction. We urge authors to be more thorough, and editors and reviewers of hydrogen storage material research to be more vigilant when assessing whether H_2_ sorption results have been adequately validated and experimental details sufficiently reported.

Despite the various issues discussed above, the field of hydrogen storage materials research remains vibrant and it will be exciting to see the further advances that can be expected in the coming years. Areas of research, such as H_2_ sorption by flexible frameworks and organic cage compounds,[^19^, ^20^] for example, have only just begun to be explored, while the study of the rich chemistry of borohydrides is a burgeoning field.^[18]^ Nanoconfining light metal hydrides in porous scaffolds also offers much promise.[^126^, ^127^] It is also worth noting that metal and complex hydrides have various other applications in hydrogen and energy storage technology, including hydrogen compression, and thermal and electrochemical energy storage;[^128^, ^129^] while both hydrides and nanoporous materials are of interest for hydrogen isotope separation.^[130]^ Accurate materials characterisation is obviously important in each case, and it is critical that such research continues with a firm focus on the reproducibility of reported results.

## Conflict of interest

The authors declare no conflict of interest.

## Biographical Information

*Darren Broom is a product manager for Hiden Isochema Ltd. He obtained his PhD in 2003 from the University of Salford, UK, and spent three years as a postdoctoral research fellow at the JRC Institute for Energy in The Netherlands, before returning to the UK to join Hiden Isochema in 2007. He is the author of Hydrogen Storage Materials: The Characterisation of Their Storage Materials, published by Springer in 2011, and is a UK representative on Task 40 “Energy storage and conversion based on hydrogen” within the Hydrogen Technology Collaboration Programme (Hydrogen TCP) of the International Energy Agency (IEA)*.

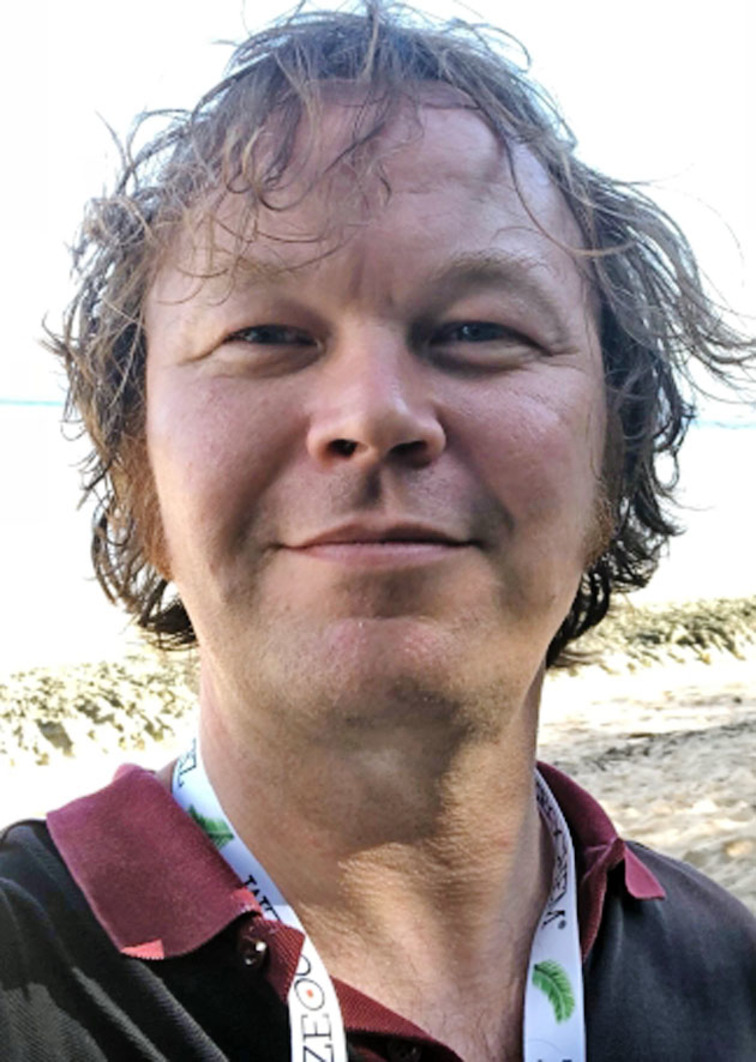

## Biographical Information

*Michael Hirscher is leading the “Hydrogen Storage Group” at the Max Planck Institute for Intelligent Systems, Stuttgart, Germany, since 1991. He studied physics at University of Stuttgart, Germany and at Oregon State University, Corvallis, USA, receiving his Dr. rer. nat. in Stuttgart 1987. In 1988 he was awarded with the Otto Hahn Medal of the Max Planck Society. Afterwards he spent a post‐doctoral fellowship at the University of Pennsylvania, Philadelphia, USA in a project with IBM Almaden Research Center, San Jose. At the International Symposium on Hydrogen & Energy in Switzerland he received the “Hydrogen & Energy Award 2015”. Since 2019 he is Task Manager of Task 40 “Energy storage and conversion based on hydrogen” within the Hydrogen Technology Collaboration Programme (Hydrogen TCP) of the International Energy Agency (IEA)*.